# Tumor-derived miR-126 promotes tumor progression through immune modulation beyond the local tumor environment

**DOI:** 10.3389/fonc.2026.1867336

**Published:** 2026-07-23

**Authors:** Takashi Akazawa, Yu Mizote, Tomoya Ekawa, Satomi Yoshida, Yachiyo Kumamoto, Yoichiro Harada, Hisataka Ogawa, Shunichi Watanabe, Shingo Hatoya, Norimitsu Inoue, Hideaki Tahara

**Affiliations:** 1 Department of Cancer Drug Discovery and Development, Research Center, Osaka International Cancer Institute, Osaka, Japan; 2 Department of Cancer Immunotherapy, Frontier Exploratory Research Domain, Next-Generation Precision Cancer Research Center, Osaka International Cancer Institute, Osaka, Japan; 3 Department of Glyco-Oncology and Medical Biochemistry, Research Center, Osaka International Cancer Institute, Osaka, Japan; 4 Institute for Glyco-core Research (iGCORE), Nagoya University, Nagoya, Japan; 5 Nitto Joint Research Department for Nucleic Acid Medicine, Research Center, Osaka International Cancer Institute, Osaka, Japan; 6 Department of Surgery, Rinku General Medical Center, Izumisano, Japan; 7 Department of Advanced Pathobiology, Graduate School of Veterinary Science, Osaka Metropolitan University, Izumisano, Japan; 8 Department of Molecular Genetics, Wakayama Medical University, Wakayama, Japan; 9 Center for Clinical Research, Osaka International Cancer Institute, Osaka, Japan

**Keywords:** immune regulation, microRNA, non-local immune modulation, regulatory T-cell (Treg), tumor immunity

## Abstract

Epidermal growth factor-like domain 7 (EGFL7) was discovered as an extracellular matrix protein with an EGF-like domain and angiogenic and vasculogenic functions. It has also been shown to play a role in immunological evasion of tumor cells through attenuation of extravasation of immune cells by reducing the expression of cell adhesion molecules on vascular endothelial cells. Furthermore, microRNA-126 (miR-126), which is encoded within the intron of *Egfl7*, has been reported to be a vasculogenic factor. However, its immune-related functions in tumors remain unclear. Here, we examined the roles of tumor-derived EGFL7 and miR-126 in *in vivo* tumor progression using *Egfl7/Mir-126* knockout and rescue tumor cell lines. Tumor growth was significantly suppressed in *Egfl7/Mir-126*-deficient cells and was restored by re-expression of miR-126, but not EGFL7. These differences in *in vivo* tumor growth were not observed in immunodeficient mice, suggesting the involvement of the adaptive immune system. CD4^+^ or CD25^+^ cell depletion suppressed the growth of miR-126-expressing tumors, whereas CD8^+^ cell depletion enhanced the growth of miR-126-deficient tumors. Histological analysis revealed an increased Foxp3^+^/CD8^+^ cell ratio in miR-126-expressing tumors during the early phase of tumor establishment. Bilateral tumor models further demonstrated that miR-126-expressing tumors promoted the growth of contralateral miR-126-deficient tumors, and a similar effect was observed using apoptotic miR-126-expressing tumor cells *In vitro* analyses using extracellular vesicles derived from dying tumor cells showed the transfer of miR-126 to CD4^+^ T cells and a higher proportion of Foxp3^+^ cells. Together, these findings suggest that tumor-derived miR-126 promotes tumor progression through non-local immune modulation, potentially involving maintenance of Treg-associated populations.

## 1 Introduction

Epidermal growth factor-like domain 7 (EGFL7) was discovered as an extracellular matrix protein possessing an EGF-like domain and has been reported as an angiogenic factor since the knockout of EGFL7 was shown to be embryonic lethal due to vascular deficiency (1–4). In addition, microRNA-126 (miR-126), encoded in the seventh intron of the *Egfl7* gene, has been shown to have multiple roles. miR-126-3p (miR-126) and miR-126-5p (miR-126^*^) are generated by the maturation of the *Mir-126* gene, which promotes the degradation of their target RNAs (5). The miR-126-3p controls other functional RNAs, including *Vegf-a* (6–8), *Spred1* (9–11), and *Pi3k2r* (12, 13), which promote or suppress angiogenesis and vasculogenesis. Thus, in the *Egfl7*/*Mir-126* gene region, these two factors act cooperatively or antagonistically on angiogenesis/vasculogenesis. This complex relationship makes it difficult to understand their roles *in vivo*. Furthermore, knockout mice lacking only *Mir-126* are embryonic lethal (9), similar to the results initially reported in mice lacking the entire *Egfl7/Mir-126* gene (3), whereas mice lacking only *Egfl7* and retaining miR-126 are not embryonic lethal (12). Although some contradictory reports exist, only miR-126, not EGFL7, appears to be responsible for angiogenesis/vasculogenesis based on these reports (14).

The EGFL7 protein expressed by tumor cells has been shown to have another function. It reduces the expression of cell adhesion molecules, such as intercellular adhesion molecule (ICAM)-1 and vascular cell adhesion molecule (VCAM)-1, on vascular endothelial cells, thereby reducing the chances of immune cells adhering to the vascular endothelial cells, leading to extravasation. In fact, the growth of tumors transfected with *Egfl7* mRNA not containing *Mir-126* was promoted by the finding that T cells remained inside the blood vessels and scarcely infiltrated into tumors (15). Based on this report, EGFL7 has been recognized as one of the factors that enable the immunological escape of tumors. Although there are some reports that both EGFL7 and miR-126 are functionally involved in angiogenesis and vasculogenesis, there are few reports on the relationship between miR-126-expressing tumors and immune responses (16).

Here, we report that tumor-derived miR-126, but not *Egfl7*, is associated with tumor progression involving non-local immune modulation. In particular, miR-126 expression in tumor cells influenced the growth of contralateral tumors in a bilateral model, suggesting effects beyond local tumor-immune system interactions. These findings indicate that tumor-derived miR-126 may contribute to intercellular immune regulation rather than exert its effects solely within tumor cells. Therefore, controlling tumor miR-126 expression may be a potential strategy to counteract tumor immune evasion.

## 2 Materials and methods

### 2.1 Mice and cell lines

Wild-type C57BL/6JJcl and BALB/cAJcl mice were purchased from CLEA Japan (Tokyo, Japan), and NOD-*scid Il2rg*^null^ (NSG) mice were purchased from Jackson Laboratory Japan (Kanagawa, Japan). OT-I (17) mice were kindly provided by Dr. W. R. Heath (The Walter and Eliza Hall Institute of Medical Research, Melbourne, Australia). All animal experiments were performed in accordance with institutional guidelines and approved by the Animal Care and Use Committee of our institute (Approval no. 22030320, 23022116, 24021909, 25030314, 26030316). All experiments involving recombinant DNA were performed in accordance with institutional guidelines and approved by the Biosafety Committee of our institute (Approval No. 20220302014, 20220302015, R5032117, R5030218, R6030615, R6030616, R7022013.R7022014, R8021713, R8021714).

The mice were maintained under specific pathogen-free conditions at the animal facility of the Osaka International Cancer Institute.

Ovalbumin (OVA)-expressing EL4 murine thymoma cells (E.G7-OVA cells) were obtained from the American Tissue Culture Collection (CRL-2113; Manassas, VA) (18). Murine colon adenocarcinoma cell Colon-26 was obtained from RIKEN BioResource Center CELL BANK (RCB2657; Ibaraki, Japan) (19, 20). E.G7-OVA cells were cultured in RPMI 1640 containing 10% fetal calf serum (FCS), 50 μM 2-mercaptoethanol, 10 mM HEPES, and 1 mM sodium pyruvate. Colon-26 cells were cultured in RPMI 1640 medium containing 10% FCS.

### 2.2 Reagents

The following antibodies and ELISA kit were used: InVivoMAb, Anti-mouse CD4 (BE0003-1, GK1.5), Anti-mouse CD8a (BE0004-1, 53-6.7), and Anti-mouse CD25 (BE0012, PC-61.5.3) for *in vivo* depletion from Bio X Cells (Lebanon, NH). Anti-mouse CD8β (ab228965, EPR22331-54, Dilution: 1:2000) and anti-FOXP3 (ab215206, EPR22102-37, 1:1000) from Abcam (Cambridge, UK) for immunohistochemistry. Anti-mouse VE-Statin Isoform 1 antibody (AF3089, Polyclonal Goat IgG, 1:1000, VE-Statin is another name of EGFL7) and Anti-Goat IgG Horseradish Peroxidase-conjugated Antibody (HAF019, 1:5000) were used for western blot analysis of EGFL7, and Mouse IFN-gamma DuoSet ELISA (DY485) was used for ELISA (R&D Systems, Minneapolis, MN).

### 2.3 Preparation of *Egfl7*/*Mir-126* knock-out and rescue cell lines

*Egfl7/Mir-126* gene was knocked out by CRISPR-Cas9 gene editing using the Guide-it CRISPR/Cas9 System (632601; TaKaRa Bio, Shiga, Japan) according to the manufacturer’s instructions. The single guide RNA (sgRNA) for *Egfl7* (Gene ID: 353156) knock-out (KO) was designed using the CHOPCHOP website (https://chopchop.cbu.uib.no/) (21), and selected three sgRNAs, sgRNA1: TGTGTCCGTCGCAAGTGGTG-agg, sgRNA2: GACGCGCTGCCTGTCTAAGG-agg, sgRNA3: TCGGGTTGATGTGCTAGAAC-agg; the last three lowercase letters are Protospacer Adjacent Motif (PAM) sequence. The sgRNA1 was cloned into the pGuide-it-ZsGreen1 vector, and the others were cloned into the pGuide-it-tdTomato vector. These vectors were transfected into tumor cells in combination with sgRNA1 and sgRNA2 (E.G7-OVA and Colon-26), using TransIT-X2 reagent (MIR600, Mirus Bio, Madison, WI), 48 h after transfection, ZsGreen1 and tdTomato double-positive transfectants were isolated using a cell sorter (S3 cell sorter, Bio-Rad Laboratories, Hercules, CA) and cloned by limiting dilution. *Egfl7/Mir-126* KO clones were screened by PCR of genomic DNA (S: 5’-TAGACCACTAGCTGACCCCATT-3,’ AS: 5’-CTAGAGACTGGTCTGGGGACAC-3’) and confirmed by quantitative PCR (qPCR) (Thermo Fisher Scientific, Foster City, CA). Another KO line of E.G7-OVA (EG7 KO.2) were prepared by in combination with sgRNA1 and sgRNA3.

*Egfl7* cDNA, common open reading frame of mRNA variants a, b, and d, was amplified by PCR using KOD Plus Neo (KOD-401, TOYOBO, Osaka, Japan), a primer pair (S: 5’-AAGAATTCACCATGTGGGGCTCCGGAGAACTGCTT-3,’ AS: 5’-TTCTCGAGTCACAGATCTTTTTTGCAGGAGCAGGA-3’), and cDNA from E.G7-OVA as a template. The PCR products were cloned into the pCR-Blunt vector and transferred into the pMXs-puro vector (RTV-012, Cell Biolabs, San Diego, CA) at the EcoRI and XhoI sites. miR expression vector was designed pMMLV-puro vector containing the EF-1α core promoter, human β-globin intron 1, murine miR-126 region (total 259 bp, including 100 bp flank sequence to both upstream and downstream of *Mir-126* gene), and human β-globin intron 2, and constructed by Vector Builder (Chicago, IL). The pMXs or pMMLV vectors were transfected into the Plat-A retroviral packaging cell line (RV-102, Cell Biolabs) using the Lipofectamine 3000 reagent (L3000, Thermo Fisher Scientific), and then, after culturing for 48 h, supernatants containing retroviral particles were collected. Cells were cultured with retroviral solution with 8 μg/mL Hexadimethrine bromide (17736-44, Nacalai Tesque, Kyoto, Japan) for 24 h, and were selected with 2 μg/mL (for E.G7-OVA) or 4 μg/mL (for Colon-26) of Puromycin Dihydrochloride (A11138, Thermo Fisher Scientific) for 7 days of culturing subsequently.

### 2.4 RNA isolation and quantitative PCR

Total RNA was prepared using the miRNeasy Micro Kit (1071023; Qiagen, Venlo, Netherlands) according to the manufacturer’s instructions. Reverse transcription (RT) was performed using the TaqMan MicroRNA Reverse Transcription Kit (4366596, Thermo Fisher Scientific) for miRNA and SuperScript IV VILO Master Mix (11756050, Thermo Fisher Scientific) for mRNA. Taqman qPCR assay was performed using Taqman Universal Master Mix II, no UNG (4440043, Thermo Fisher Scientific), and TaqMan MicroRNA Assay (hsa-miR-126 (Assay ID: 002228), hsa-miR-126* (000451) (common to humans and mice), U6 snRNA (001973)) or TaqMan Gene Expression Assay ((*Egfl7* (Mm00618004_m1) or *Gapdh* (4352339E)). qPCR was performed using Applied Biosystems Quant Studio 3 (Thermo Fisher Scientific). The relative expression values were calculated by comparative Ct method (2−ΔΔCt, ddCt). Results were calculated using the endogenous control U6 snRNA for microRNAs, *Gapdh* for *Egfl7*; samples with Ct values ≥ 45 were excluded from the analysis as ND (not detected).

### 2.5 *In vitro* assays

Cell growth analysis was performed using Cell Counting Kit-8 reagent (Dojindo Laboratories, Kumamoto, Japan), and absorbance was measured at 450 nm, according to the manufacturer’s protocol. OT-I splenocytes were prepared using Lympholyte M (CL5035; Cedarlane, Burlington, Canada). For detection of IFN-gamma using ELISA. Splenocytes were cultured with 3 × 10^3^ manipulated-EG7 cells at a ratio of 20:1 or 50:1 for 72 h in V-bottomed 96-well plates (Corning, Corning, NY) in a total volume of 200 μL.

Apoptotic bodies were prepared by incubating 10^7^ manipulated E.G7-OVA cells for 12 h after irradiation at 90 Gy (22), and the culture supernatant was centrifuged at 800 × g for 10 min. The supernatant was centrifuged at 16,000 × g for 20 min to collect the precipitate, and total RNA was isolated (23).

For the miR-126 transfer assay, Naive CD4^+^ T cells were isolated from the spleen and thymus using the EasySep™ Mouse Naive CD4^+^ T Cell Isolation Kit (ST-19765; STEMCELL Technologies, Vancouver, Canada). These T cells were incubated with apoptotic bodies collected from EG7 KO or rescued (RQ) cells at a 25-fold excess of cell numbers for 6 h; then, T cells were washed and total RNA was isolated. For the phenotypic analysis of CD4^+^ T cells, CD4^+^ T cells were isolated from the spleen using CD4 (L3T4) MicroBeads (130-117-043; Miltenyi Biotec, Bergisch Gladbach, Germany). The cells were incubated with apoptotic bodies collected from EG7 KO or RQ cells at a 25-fold excess of cell numbers for 6 h, and then cultured with Dynabeads™ Mouse T-Activator CD3/CD28 for T-Cell Expansion and Activation (11452D; Thermo Fisher Scientific), and 50 U/mL Human IL-2 Recombinant Protein (200-02; Thermo Fisher Scientific) for 72 h.

### 2.6 *In vivo* mouse models

On day 0, E.G7-OVA (10^6^) was subcutaneously inoculated into the right back of 8–12-week-old female C57BL/6 mice or 8–10-week-old female NSG mice. Colon-26 cells (5 × 10^5^) were subcutaneously inoculated into the right back of 8–10-week-old female BALB/c mice. Female mice were used throughout the study to maintain consistency across experiments. When transplantation was performed on both backs, 10^6^ cells were transplanted on the right side, and the indicated number of cells was transplanted on the left side within 30 min. To prepare dying cells, manipulated E.G7-OVA cells were irradiated with 90 Gy of X-rays and then transplanted into mice within 2 h. Tumor volume was calculated using the following formula: tumor volume (cm^3^) = long diameter (cm) × short diameter (cm) × short diameter (cm) × 0.4. The mice were euthanized according to the approved humane endpoint criteria. In this study, a tumor burden corresponding to more than 10% of the body weight was used as an endpoint, and animals meeting this criterion were euthanized at the next scheduled measurement. In *in vivo* depletion assays, 250 μg of antibodies were injected intraperitoneally on day -1.

### 2.7 Immunohistochemistry and analysis

Immunohistochemical staining with 3,3’-diaminobenzidine (DAB) was performed automatically using BOND-RX (Leica Biosystems, Wetzlar, Germany) and BOND Polymer Refine Detection (DS9800, Leica Biosystems) with antibodies according to the manufacturer’s instructions. Whole images of the specimens were acquired using a SLIDEVIEW VS200 (Olympus Scientific Solutions, Tokyo, Japan). ImageJ software (https://imagej.nih.gov/ij/) was used to quantify the tumor area (24). One window (0.2 mm^2^) was randomly set per 1 mm^2^ of tumor area, and the number of CD8 and Foxp3 positive cells was counted. The number of cells within the window was summed to calculate the number of cells per 1 mm^2^.

### 2.8 Western blot analysis

Cell lysates were prepared using RIPA Lysis Buffer containing Halt Protease Inhibitor Cocktail (1862209, Thermo Fisher Scientific) and 5 mM EDTA. Cell lysates (4.5 × 10^5^ cells/lane) were separated by SDS-PAGE (4–20% Mini-PROTEAN TGX Gels, 4561096, Bio-Rad Laboratories) and transferred to an Immobilon-P membrane (IPVH07850, Merck Millipore, Darmstadt, Germany). The membrane was stained with an Anti-mouse VE-Statin Isoform 1 antibody, followed by an anti-goat IgG horseradish peroxidase-conjugated secondary antibody. Detection was performed using Clarity Western ECL Substrate (1705060; Bio-Rad Laboratories) and LuminoGraph (ATTO, Tokyo, Japan).

### 2.9 Flow cytometry analysis

The proportion of CD25^+^ Foxp3^+^ cells in *in vitro* cultures was measured using a spectral cell analyzer (Sony Biotechnology, San Jose, CA), and the data were analyzed using FlowJo software (BD Biosciences, Ashland, OR). The following reagents were purchased from BioLegend (San Diego, CA): Zombie NIR Fixable Viability Kit (cat. no. 423106), TruStain FcX™ (anti-mouse CD16/32) Antibody (cat. no. 101320), fluorescein isothiocyanate (FITC)-conjugated anti-mouse CD4 (cat. no. 100406) antibody, allophycocyanin (APC)-conjugated anti-mouse CD25 antibody (cat. no. 102012), phycoerythrin (PE)-conjugated anti-mouse CD152 antibody (CTLA-4; cat. no. 106306), a FOXP3 Fix/Perm Buffer Set (cat. no. 421403), BV421 anti-mouse FOXP3 antibody (cat. no. 126419), a BV421-conjugated rat IgG2b isotype control (cat. no. 400655), and PE-conjugated Armenian hamster IgG isotype control (cat. no. 400908). The cells were first treated with Zombie NIR Fixable Viability Reagent, followed by Fc blocking and surface antibody staining. The cells were then fixed and permeabilized using the FOXP3 Fix/Perm Buffer Set according to the manufacturer’s instructions, followed by intracellular staining with an anti-FOXP3 antibody. The BV421-conjugated rat IgG2b isotype control and PE-conjugated Armenian hamster IgG isotype control were used as isotype controls.

### 2.10 Statistical analysis

The data are expressed as the mean ± SD. GraphPad Prism 9 (GraphPad Software, San Diego, CA) was used for statistical analysis. The significance of the differences between groups was analyzed using Welch’s *t*-test (comparison of two groups), or 2-way ANOVA with Dunnett’s test or Tukey’s test (multiple comparisons [more than three groups]). Two-sided *P*-values <.05 were considered statistically significant.

## 3 Results

### 3.1 *Mir-126* but not *Egfl7* lacking tumor show growth retardation *in vivo*

**Figure 1:**
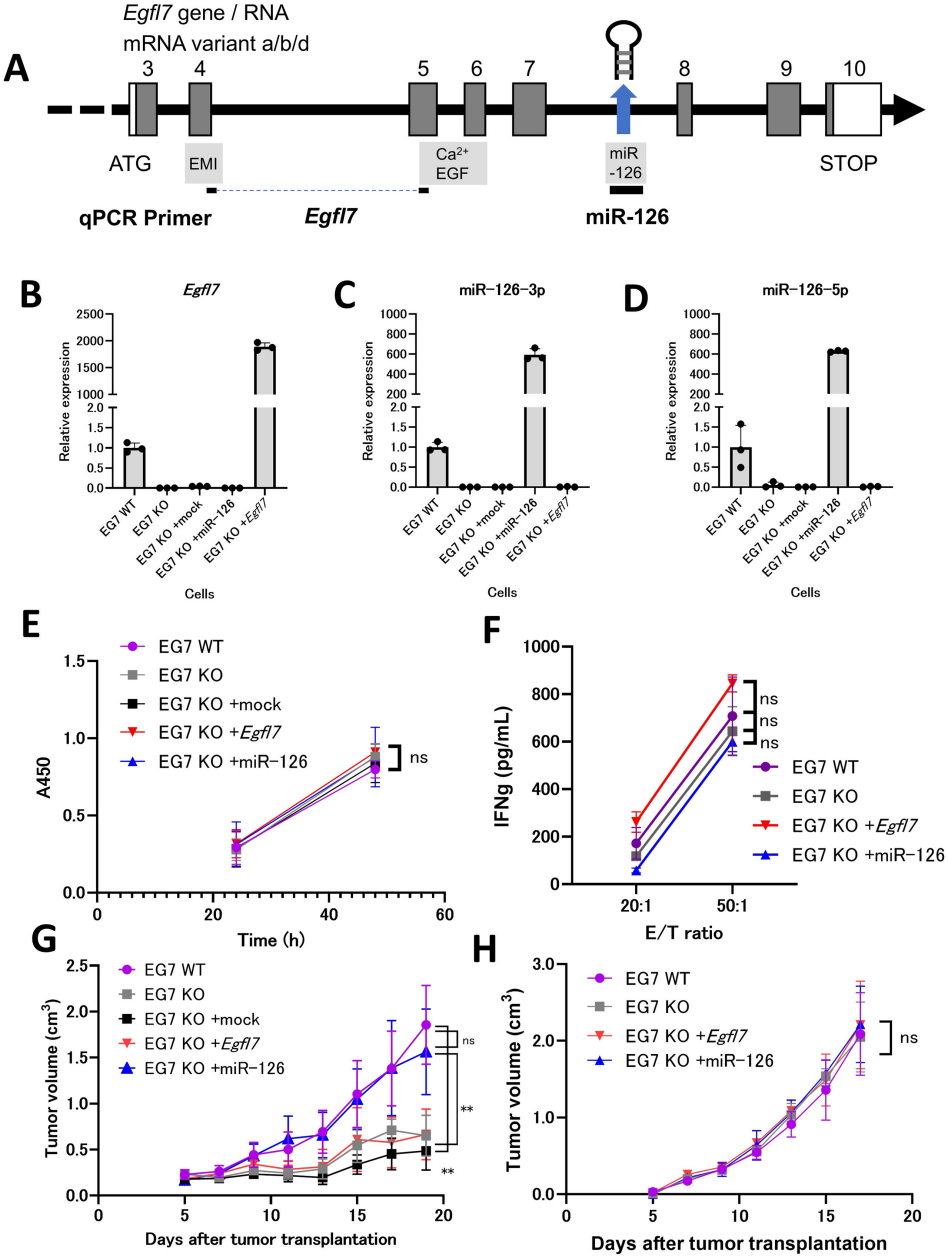
*Mir-126* but not *Egfl7* lacking tumor show tumor growth retardation in *in vivo*. **(A)** Structure of the *Egfl7* gene including *Mir-126*. Blue arrow: *Mir-126* encoding region. Bar: quantitative PCR (qPCR) primer for detection. Expression levels of target genes in E.G7-OVA WT, KO, and *Egfl7* or *Mir-126* restored rescued cell lines (RQ), **(B)** *Egfl7*, **(C)** miR-126-3p, and **(D)** miR-126-5p. The relative expression levels of *Egfl7* and miR-126 were calculated using the ddCt method based on *Gapdh* and U6 snRNA, respectively. **(E)** *In vitro* growth of WT, KO, and RQ cell lines using a cell counting kit (n = 8). **(F)** IFN-γ production by OT-I splenocytes against WT, KO, and RQ cell lines based on responses to OVA peptide, SIINFEKL, bound to H-2K^b^ (n = 3). *In vivo* tumor growth after transplanting 10^6^ cells of WT, KO, or RQ cell lines subcutaneously into the right back of **(G)** immunocompetent C57BL/6 mice or H) immunodeficient NSG mice (n = 6). The data are shown as means ± SD. ns: not significant, **p < 0.01. (**(E, F)** Dunnett’s test vs. EG7 WT; **(G, H)** Tukey’s test).

To examine the functions of *Egfl7* and miR-126 in an *in vivo* mouse tumor transplantation model with the E.G7-OVA (OVA gene transfectant of murine thymoma cell line EL4), *Egfl7* and *Mir-126* expression-lacking cells (EG7 KO) were prepared using the CRISPR-Cas9 system based on gene structures (**Figure 1A**). Next, we prepared the rescued cells (RQ), which EG7 KO cells genetically modified to continuously express miR-126 or *Egfl7*. The expression of each gene was confirmed by qPCR (**Figures 1B–D**). Expression analysis confirmed markedly reduced *Egfl7*, miR-126-3p, and miR-126-5p levels in KO cells and their re-expression in the corresponding RQ cells, with miR-126 levels being higher in RQ cells than in WT cells. EG7 WT, KO, miR-126 RQ cells (EG7 KO +miR-126), and EGFL7 RQ cells (EG7 KO +*Egfl7*) showed similar proliferation rates *in vitro* (**Figure 1E**) and similar responses based on OVA antigen, measured with IFN-γ production by OT-I splenocytes (**Figure 1F**) even though those by WT splenocytes were undetectable level. However, miR-126 RQ cells but not EGFL7 RQ cells showed recovered *in vivo* tumor growth rates in immunocompetent C57BL/6 mice (**Figure 1G**). When these tumors were transplanted into immunodeficient NSG mice, their growth rates did not differ significantly (**Figure 1H**). These data indicate that some immunological responses, except for *in vitro* direct interactions based on antigens, might be responsible for the differences in tumor growth *in vivo*. Additional rescue cell experiments were performed using the EG7 KO.2 line and similar differences of *in vivo* tumor growth were observed among WT, KO and RQ cells (**Supplementary Figures 1A–D**). KO and RQ cells were also established from BALB/c-derived Colon-26 tumor and showed similar differences in *in vivo* tumor growth (**Supplementary Figures 2A–D**). These findings indicated that miR-126, rather than EGFL7, is more closely associated with the observed differences in tumor growth *in vivo*. Thus, rescue experiments should be interpreted as demonstrating the functional relevance of miR-126 re-expression in this model, rather than strict physiological rescue.

### 3.2 miR-126-expressing tumors show rapid growth *in vivo* in association with impaired CD8^+^ T cell–mediated antitumor responses

**Figure 2:**
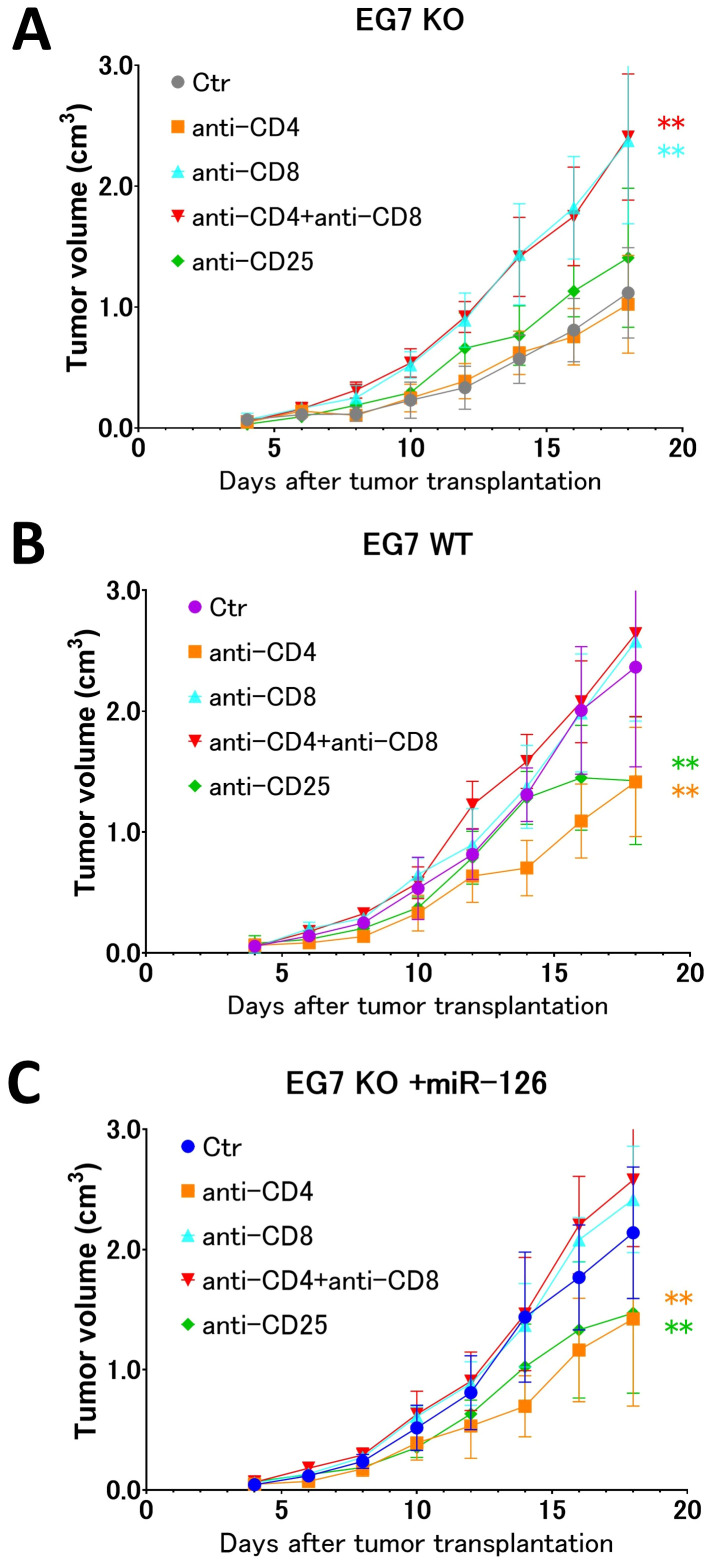
miR-126-expressing tumors show rapid growth *in vivo* in association with impaired CD8^+^ T cell–mediated antitumor responses (E.G7-OVA/C57BL/6). Effect of depletion of CD8^+^, CD4^+^, or CD25^+^ cells by antibody treatment on tumor growth compared with the non-treatment control (Ctr). On the day before tumor transplantation, mice were administered with indicated antibodies intraperitoneally (250 microgram/head) to deplete immune cells, including CTLs and CD25^+^ Treg-associated populations. E.G7-OVA **(A)** KO, **(B)** WT, **(C)** RQ (restored miR-126) were transplanted subcutaneously into the right back of C57BL/6 mice (n = 6). The data are shown as means ± SD. **p < 0.01 vs Ctr (Dunnett’s test vs. Ctr).

To identify the immune cells that affect tumor growth rates (**Figure 2**), cell depletion experiments *in vivo* were performed using antibodies. Regarding EG7 *Egfl7*/*Mir-126* KO tumors, depletion of CD8^+^ cells, mainly CTLs, alone, or both CD4^+^ and CD8^+^ cells, showed a strong increase in tumor size compared to the untreated control group. However, the depletion of CD4^+^ or CD25^+^ cells alone had no effect on KO tumor growth (**Figure 2A**). CD8^+^ CTLs are significantly involved in the regression of KO tumors. In contrast, depletion of CD4^+^ or CD25^+^ but not CD8^+^ cells led to strong suppression of EG7 WT tumor growth; however, tumor growth suppression was not observed when both anti-CD4 and anti-CD8 antibodies were administered (**Figure 2B**). Furthermore, EG7 RQ tumors showed similar tumor growth suppression by CD4^+^ or CD25^+^ cell depletion, as observed in EG7 WT tumor-bearing mice (**Figure 2C**). Together, the suppression of tumor growth by CD4^+^ or CD25^+^ cell depletion, and the loss of this effect after concomitant CD8^+^ cell depletion, suggest that CD4^+^ CD25^+^ Treg-associated populations may suppress CD8^+^ CTL-mediated antitumor responses in miR-126-expressing tumors.

### 3.3 Foxp3^+^ cells appear predominantly in miR-126-expressing tumors in the early stages

**Figure 3:**
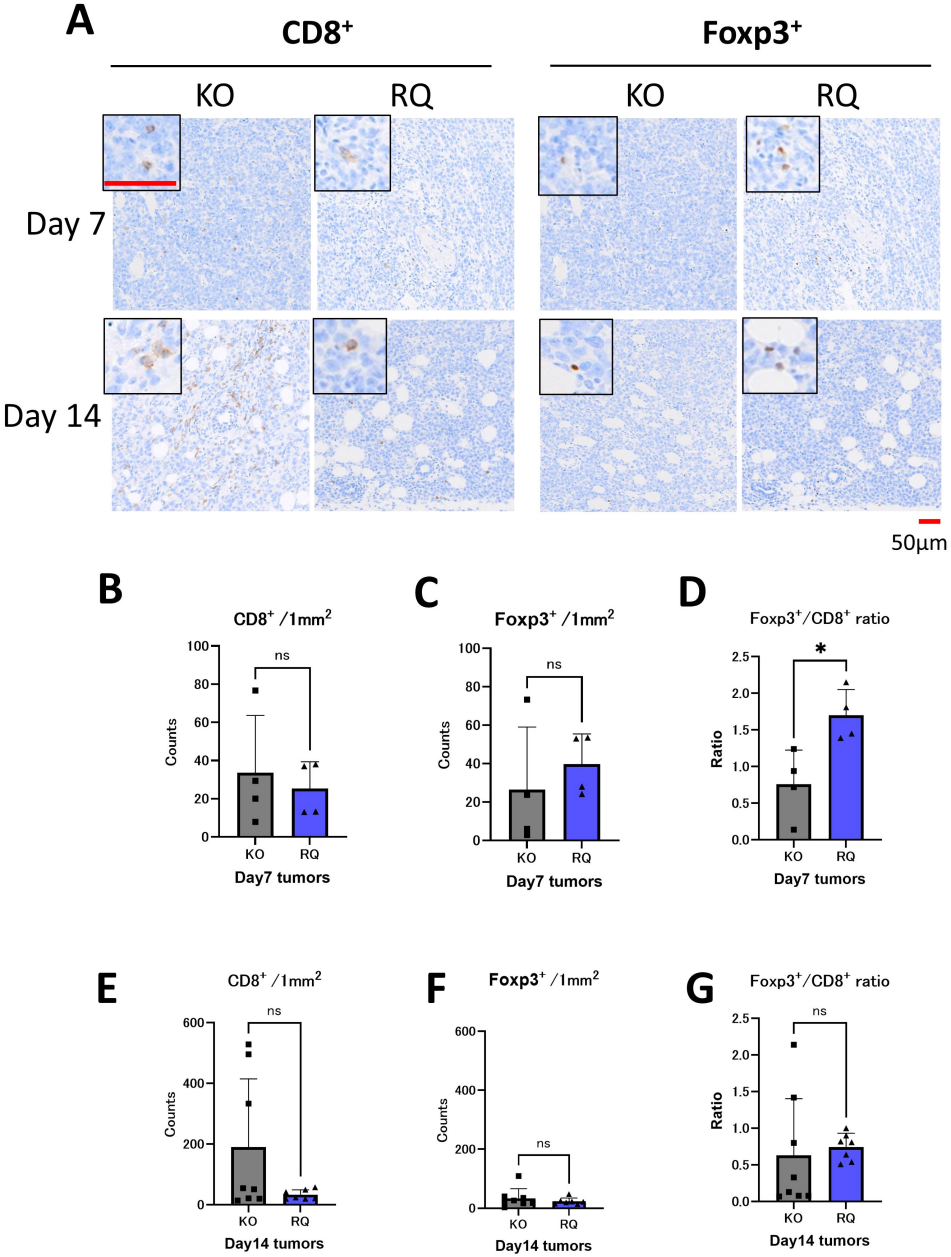
Foxp3^+^ cells appear predominantly in miR-126-expressing tumors in the early stages. **(A)** Immunohistochemical staining of CD8 and Foxp3 positive cells in EG7 KO and RQ tumors on day 7 and day 14 after transplantation. The average counts of CD8^+^ **(B)** and Foxp3^+^ **(C)** cells, and Foxp3^+^/CD8^+^ cell ratio **(D)** in 1 mm^2^ tumors on day 7 (n = 4). The average counts of CD8^+^ **(E)** and Foxp3^+^ **(F)** cells, and Foxp3^+^/CD8^+^ cell ratio **(G)** in 1 mm^2^ tumors on day 14 (n = 7-8). A window (about 0.2 mm^2^) was randomly allocated for every 1 mm^2^ of tumor to count the number of cells (see **Supplementary Figure 3**). The data are shown as means ± SD. *p < 0.05 (Welch’s *t*-test).

To examine the ratio of regulatory T-cells (Tregs; Foxp3^+^) and CTLs (CD8^+^) in tumor infiltrating lymphocytes (TILs) of miR-126-lacking (KO) and -expressing (RQ) tumors, immunohistochemical staining was performed on day 7 tumors (no change in *in vivo* tumor size between KO and RQ) and day 14 tumors (RQ is bigger than KO) (**Figure 3A**; **Supplementary Figure 3**). On day 7, although the mean numbers of CD8^+^ and Foxp3^+^ cells in whole tumors were not significantly different between KO and RQ tumors, the Foxp3^+^/CD8^+^ ratio was significantly higher in RQ tumors than in KO tumors (**Figures 3B–D**). On day 14, no such differences were observed (**Figures 3E–G**).

### 3.4 Non-local immune modulation associated with miR-126-expressing tumors

**Figure 4:**
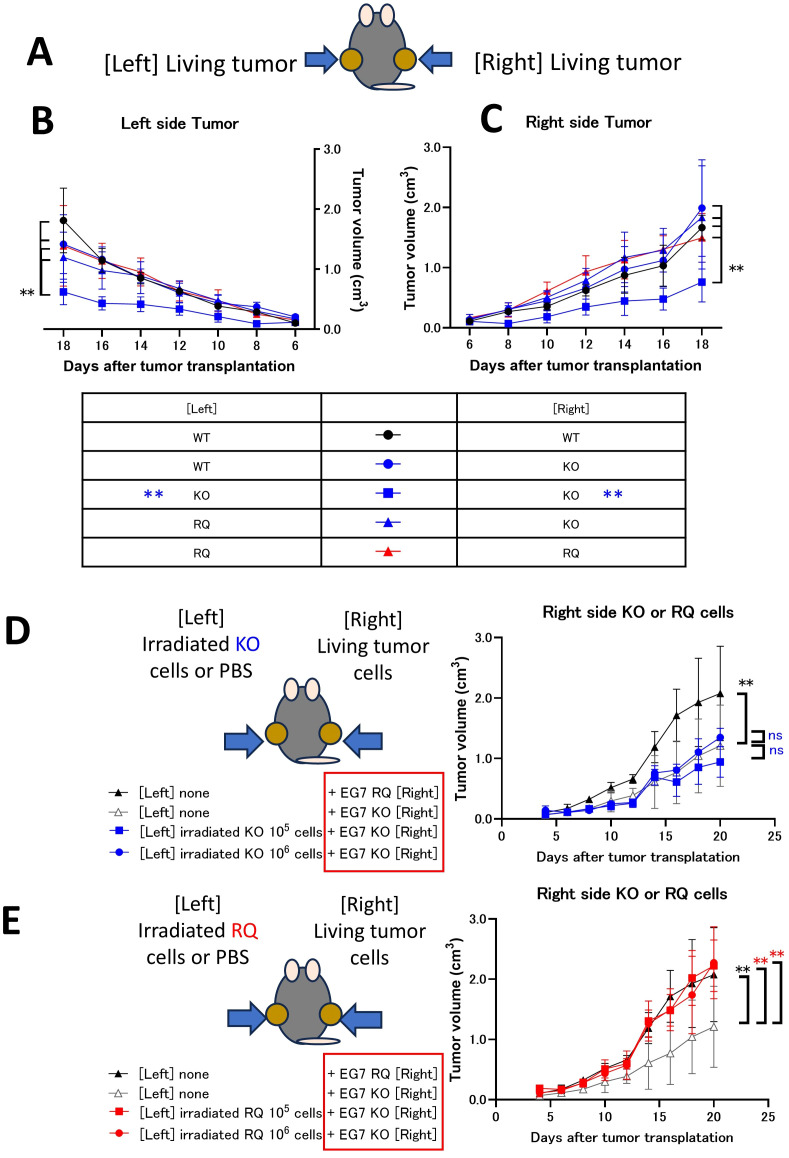
Non-local immune modulation associated with miR-126-expressing tumors. *In vivo* tumor growth was observed when different combinations of tumors were transplanted onto both sides of the back of the mouse. **(A)** Schematic of the mouse transplantation model, **(B)** left-side tumors, and **(C)** right-side tumors. Symbols indicate the left-side tumors (circle, EG7 WT; triangle, EG7 RQ; square, EG7 KO), whereas colors indicate the right-side tumors (black, EG7 WT; blue, EG7 KO; red, EG7 RQ). A total of 10^6^ tumor cells were subcutaneously transplanted into the backs of C57BL/6 mice (n = 6). The data are shown as means ± SD. **p < 0.01 vs WT tumor on each side of WT-WT group (Dunnett’s test). *In vivo* EG7 KO tumor growth on the right side when irradiated EG7 KO cells (**(D)** blue) or RQ cells (**(E)** red) were subcutaneously administered to the left back of C57BL/6 mice (n = 5). Circle: irradiated 10^6^ cells; square: 10^5^ cells. Closed black triangle: EG7 RQ tumor growth on the right side without administration on the left side; open gray triangle: EG7 KO tumor growth on the right side without administration on the left side. **p < 0.01 vs the group of EG7 KO tumor on the right side without administration on the left side (Dunnett’s test).

Next, we investigated whether non-local immune modulation influencing *in vivo* tumor growth could be induced by the miR-126-expressing tumors. The miR-126 non-expressing EG7 KO cells were transplanted into the right back of the mice, and simultaneously, EG7 KO cells or miR-126-expressing EG7 WT or RQ cells were transplanted into the left back. When EG7 KO cells were transplanted on both sides, both tumors grew at the same rates, which were comparable to the growth rates of EG7 KO tumors transplanted alone, whereas the tumor growth rates of EG7 KO tumors were accelerated when miR-126-expressing tumors were transplanted on the left side (**Figures 4A–C**). In contrast, transplantation of KO cells did not affect the growth rate of miR-126-expressing cells on the opposite side. These results suggest that miR-126 expressed by tumor cells contributes to the local tumor microenvironment and non-local modulation of tumor growth. One of the pathways for miR-126 transfer has been reported to be the one through apoptotic bodies (25, 26). To examine whether apoptotic cells were involved in miR-126-related immune-regulation, we administered irradiated RQ or KO cells on the left side instead of living cells. When the irradiated KO cells were administered to the left side, the proliferation rate of living KO cells on the right side did not change (**Figure 4D**). However, when irradiated RQ cells were administered on the left side, the proliferation of KO cells on the right side significantly increased (**Figure 4E**). These results suggest that nonlocal immune modulation is mediated by apoptotic miR-126-expressing cells.

### 3.5 Dying tumors release apoptotic bodies containing microRNAs and transfer to T cells

Based on the results shown in **Figures 4D** and **E**, we investigated whether tumor-derived miR-126 was transferred to naïve T cells via apoptotic bodies, thereby affecting the immune response (**Supplementary Figure 4A**) (22). EG7 RQ and KO cells (10^7^ cells) were irradiated *in vitro* to induce apoptosis. After irradiation, apoptotic bodies were collected by differential centrifugation and the amount of miR-126 contained within the apoptotic bodies was measured. Similar amounts of total RNA were collected from the apoptotic bodies derived from both cell types. However, the relative content of miR-126 in the apoptotic bodies from RQ cells was significantly higher than that from KO cells (**Supplementary Figures 4B, C**). When apoptotic bodies from RQ cells were added to the culture of naïve T cells for 6 h, miR-126-3p and 5p were increased in T cells compared with no treatment or treatment with apoptotic bodies from KO cells (**Supplementary Figures 4D, E**). These results suggested that miR-126 in the apoptotic bodies of tumor cells might be transferred to T cells.

### 3.6 Apoptotic bodies prepared from miR-126-expressing tumor cells are associated with the maintenance of Foxp3^+^ cells within the CD4^+^ T cell population *in vitro*

We examined whether miR-126 transferred via apoptotic bodies influences CD4^+^ T cell phenotypes. Isolated CD4^+^ T cells were exposed to apoptotic bodies derived from EG7 KO or RQ cells and then stimulated with CD3/CD28 beads in the presence of IL-2 (**Supplementary Figure 5A**). Flow cytometric analysis showed that treatment with RQ-derived apoptotic bodies resulted in a significantly higher proportion of Foxp3^+^ cells among CD4^+^ T cells than treatment with KO-derived apoptotic bodies or the control condition, suggesting possible maintenance, including relative preservation, of the CD25^+^Foxp3^+^ cell population under these culture conditions (**Supplementary Figures 5B–D**). The expression level of CTLA-4, one of the representative markers of activated/effector Tregs, was not significantly altered under these conditions (**Supplementary Figure 5E**).

## 4 Discussion

EGFL7, an extracellular matrix protein, plays a role not only in vasculogenic function but also in tumor immune escape by attenuating immune cell extravasation. The miR-126, encoded within the intron of *Egfl7*, is also a vasculogenic factor; however, its immune-related functions have not been fully investigated. In this study, we showed that tumor miR-126 is associated with non-local immune modulation beyond the local tumor environment, potentially contributing to the suppression of antitumor immunity.

Experiments on tumor transplantation models using miR-126 KO and RQ in immunocompetent and immunodeficient mice (**Figures 1G, H**) revealed that miR-126 expression by tumor cells affected the tumor growth rate via the adaptive immune system in mice *in vivo.* These findings suggest that this effect is mediated by immune mechanisms, rather than by miR-126 acting solely within tumor cells. In immune cell depletion experiments, CD4^+^CD25^+^ T cells populations in mice with tumors expressing miR-126 might negatively regulate the function of CD8^+^ CTLs and promote tumor progression (**Figure 2**). CD8^+^ T cells are likely to be involved in tumor control, although their functional status was not directly examined in the present study. In contrast, EGFL7 protein expression was detected in RQ but not WT and KO cells (**Supplementary Figures 6A, B**), but EGFL7 expression did not significantly affect the growth of the parental *Egfl7/Mir-126* KO cells *in vivo* (**Figure 1G**; **Supplementary Figure 2D**). These results indicated that miR-126, rather than EGFL7, was associated with immunosuppression in our tumor model. However, because tumor vascular density was not directly evaluated in this study, angiogenesis-related contributions could not be completely excluded. It should be noted that CD25^+^ populations include not only regulatory T cells, but also activated conventional CD4^+^ and CD8^+^ T cells. Therefore, these results reflected Treg-associated populations, rather than definitively identifying Foxp3^+^ Tregs.

Tregs have been shown to play a significant role in tumor immune escape. In particular, intratumoral Treg infiltration observed on histological examination is closely associated with tumor progression, patient prognosis, and response to various therapies (27–31). These observations have been attributed to the tumor microenvironment, which is influenced by multiple immunosuppressive functions of Tregs, including the release of humoral factors (32) and regulation via direct contact (33, 34). Clarifying the role of Foxp3^+^ cell infiltration and the balance between Foxp3^+^ cells and CTLs remains important during the early stages of tumor development. In the current study, we examined the histological characteristics of TILs by immunostaining with anti-Foxp3 and anti-CD8 monoclonal antibodies. As shown in **Figure 3**, the Foxp3^+^/CD8^+^ ratio was significantly higher in miR-126-expressing RQ tumors on day 7 post-transplantation, but not on day 14. Although not statistically significant, some KO tumors showed relatively high levels of CD8^+^ cell infiltration on day 14. Nevertheless, the difference in the Foxp3^+^/CD8^+^ ratio observed on day 7 was no longer evident at the later time points. These temporal differences suggest that miR-126-associated immunoregulation may be particularly relevant during the early phase of tumor establishment. A single administration of an anti-CD4 or anti-CD25 antibody on day -1 was designed to assess the contribution of pre-existing CD25^+^/Treg-associated populations during early tumor establishment, based on a previous report (35), rather than to prevent the induction or recruitment of lymphocytes that emerge during later tumor progression. CD4 depletion has also been investigated as an antitumor immunological approach (36). The focus on pre-existing CD25^+^/Treg-associated populations is conceptually consistent with naturally occurring Tregs and their maintenance (37). Inhibition of tumor growth following antibody treatment supports the possible involvement of preexisting CD25^+^/Treg-associated populations in the early phase of tumor establishment. Further functional characterization is required to elucidate the effects of CD25^+^ Treg-associated populations on the activation or exhaustion of tumor-infiltrating CD8^+^ T cells.

Furthermore, the bilateral tumor model showed that miR-126 affects not only the local tumor microenvironment, but also tumor growth at distant sites (**Figure 4**), suggesting effects beyond local tumor-immune system interactions. Tumor miRNAs are generally known to regulate target mRNAs within the same cell (5, 38). Accumulating evidence suggests that miRNAs can be transferred between cells via apoptotic bodies or extracellular vesicles (25, 26, 39, 40). Vascular endothelial cells undergoing apoptosis have been shown to release apoptotic bodies containing miR-126 and transfer them to neighboring cells (25, 26). In addition, miRNAs can be transferred to surrounding or specific cells, including T cells, via extracellular vesicles released from tumor cells (39, 40). Indeed, we confirmed the transfer of miR-126 from apoptotic bodies to CD4^+^ T cells (**Supplementary Figure 4**). These observations support the possibility that tumor-derived miR-126 acts on recipient cells through intercellular transfer. Phenotypic analysis of CD4^+^ T cells after treatment with apoptotic bodies showed that the proportion of Foxp3^+^ cells among CD4^+^ T cells was higher following exposure to miR-126-containing apoptotic bodies than after exposure to apoptotic bodies lacking miR-126 (**Supplementary Figure 5**). This suggests that miR-126 transferred by apoptotic bodies is involved in the relative preservation of the Foxp3^+^ cell population. This is consistent with previous reports showing that endogenous miR-126 in Tregs contributes to the maintenance of Foxp3 expression (41, 42). However, this assay models only local interactions; therefore, these findings should be considered supportive evidence and do not fully explain the non-local effects observed *in vivo*. In addition, among the Treg-associated markers, CTLA-4 expression was evaluated (**Supplementary Figure 5E**). Although CTLA-4 is a representative marker of activated/effector Tregs (43), its expression was not significantly altered after exposure to miR-126-containing apoptotic bodies. Therefore, the increased proportion of Foxp3^+^ cells was not accompanied by evidence supporting the induction of the CTLA-4^high^ activated/effector Treg phenotype. It is possible that Treg functional changes occur through mechanisms that are not reflected by CTLA-4 expression alone. However, the effects of miR-126-containing apoptotic bodies on the suppressive activity and other functional properties of Tregs remain unclear. An investigation of multiple aspects, including the importance of miR-126-3p and 5p, target molecules, and mechanistic pathways, will provide a better understanding of the role of miR-126 and novel strategies for cancer therapy.

In human studies, it has been reported that miR-126 is a good prognostic factor for tumor growth suppression (44, 45), or a predictive marker for anti-VEGF antibody treatment (46). These correlations between higher blood miR-126 concentrations (or tumor miR-126 expression) and good prognosis in humans are somewhat contradictory to our findings in *in vivo* models that miR-126-deficient tumors showed tumor growth suppression without increasing Tregs, predicting a better prognosis. Since previous miRNA measurements were performed on samples extracted from whole blood, if only miRNAs contained in extracellular vesicles, such as exosomes (47) or apoptotic bodies, are functional, then detecting only miRNAs contained in extracellular vesicles might allow different conclusions to be drawn from the existing results.

In this study, we found that tumor miR-126 might be associated with immune modulation beyond local tumor effects and tumor growth, potentially involving Foxp3^+^ cell infiltration, particularly in the early stages of tumor formation. The transfer of miR-126 from tumors to T cells via apoptotic bodies may contribute to the maintenance of Foxp3^+^ cell populations, which may be related to the immune modulation beyond the local tumor environment observed in the bilateral tumor model. Further studies on the regulatory mechanisms of tumor miR-126 expression and release, along with its roles in T cells, may lead to the development of novel strategies to control tumor immunological evasion.

## Data Availability

The original contributions presented in the study are included in the article/**Supplementary Material**. Further inquiries can be directed to the corresponding author.

## Supplementary material

Supplementary Material for this article was uploaded separately (1 document file including 6 figures).
