# Supplementary material for: Tumor-derived miR-126 promotes tumor progression through immune modulation beyond the local tumor environment
Source: Front Oncol. 2026 Jul 23;16:1867336. doi: 10.3389/fonc.2026.1867336 (PMC13441819; doi:10.3389/fonc.2026.1867336)
Supplement: Supplementary file 1 [file DataSheet1.docx]

Supplementary Material

**Tumor-derived miR-126 promotes tumor progression through immune modulation beyond the local tumor environment**

Takashi Akazawa^1,2,*^, Yu Mizote^1,2^, Tomoya Ekawa^1,2^, Satomi Yoshida^1,2^, Yachiyo Kumamoto^1,2^, Yoichiro Harada^3,4^, Hisataka Ogawa^5^, Shunichi Watanabe^1,6^, Shingo Hatoya^6^, Norimitsu Inoue^7^ and Hideaki Tahara^1,8^

*** Correspondence: Takashi Akazawa, akazawa-ta@oici.jp**

# 1 Supplementary Figures and Tables

## 1.1 Supplementary Figures

**Supplementary Figure S1.** *Mir-126* but not *Egfl7* lacking tumor show tumor growth retardation in *in vivo* (another KO line, EG7 KO.2 /C57BL6). Expression levels of target genes in E.G7-OVA WT, KO.2, and *Egfl7* or *Mir-126* restored rescue cell lines, A) *Egfl7*, B) miR-126-3p, C) miR-126-5p. The relative expression levels of *Egfl7* and miR-126 were calculated using the ddCt method based on *Gapdh* and U6 snRNA, respectively. D) *In vivo* tumor growth after transplanting 10^6^ cells of WT, KO, or rescue cell lines subcutaneously into the right back of immunocompetent C57BL/6 mice (n = 6). The data are shown as means ± SD. ns: not significant, ***p* <0.01 (Tukey’s test).

**Supplementary Figure S2.** miR-126-expressing tumors show rapid tumor growth *in vivo* (Colon-26/BALB/c). Expression level of target genes in Colon-26 WT, KO, and the *Egfl7* or *Mir-126* restored rescue cell lines by qPCR, A) *Egfl7*, B) miR-126-3p, C) miR-126-5p. The relative expression levels of *Egfl7* and miR-126 were calculated using the ddCt method based on *Gapdh* and U6 snRNA, respectively. D) *In vivo* tumor growth of Colon-26 WT, KO, or rescue cell lines in BALB/c mice (n = 6). Tumor cells (5 × 10^5^) were transplanted subcutaneously into the right back of the immunocompetent mice. The data are shown as means ± SD. ns: not significant, **p* < 0.05, ***p* < 0.01 (Tukey’s test).

**Supplementary Figure S3.** Analysis procedure for tumor-infiltrating Foxp3^+^ cells and CD8^+^ cells. The tumor area in each section was calculated based on the scale bar using ImageJ software. The number of positive cells in the images was manually counted by randomly setting a 0.2 mm^2^ window per 1 mm^2^.

**Supplementary Figure S4.** Dying tumors release apoptotic bodies containing microRNAs and transfer T cells. A) Experimental procedures and samples. Target gene expression in apoptotic bodies isolated from 10^7^ cells equivalent by qPCR, B) Relative content of miR-126-3p, and C) miR-126-5p, in apoptotic bodies of EG7 KO and RQ cells. The collected apoptotic bodies from 10^7^ cells of KO and RQ cells contained 297 and 213 ng of total RNA, respectively. The data are shown as means ± SD (n = 3). ***p* < 0.01, ****p* <0.001 (Welch’s *t*-test). D) Relative content of miR-126-3p, and E) miR-126-5p in naïve T cells incubated with the apoptotic bodies collected from EG7 KO or RQ cells at a 25-fold excess of cell numbers. NO: no treatment. The data are shown as means ± SD (n = 4). **p* <0.05 (Tukey’s test).

**Supplementary Figure S5.** Apoptotic bodies prepared from miR-126-expressing tumor cells are associated with the maintenance of Foxp3⁺ cells within the CD4⁺ T cell population *in vitro*. A) Experimental procedures and samples. B) Representative gating strategy for flow cytometric analysis. Dead cells and doublets were excluded and CD4⁺ cells were gated prior to the identification of CD25⁺ Foxp3⁺ cells. Foxp3 positivity was determined using an isotype control. C) Representative flow cytometry plots showing the proportion of CD25⁺ Foxp3⁺ cells and CTLA-4 expression in CD4⁺ cells exposed to apoptotic bodies derived from EG7 KO or RQ cells. D) The proportion of CD25⁺ Foxp3⁺ cells among CD4⁺ cells. E) CTLA-4 geometric mean fluorescence intensity (GMFI) in CD25⁺ Foxp3⁺ cells. Apoptotic bodies were collected from EG7 KO or RQ cells and added at a 25-fold excess relative to the number of CD4^+^ T cells. NO, no treatment. Data are shown as means ± SD (n = 3). ***p* < 0.01, ****p* < 0.001 (Tukey’s test).

**Supplementary Figure S6.** The comparison of EGFL7 protein expression in cell lines used in this experiment. Western blot analysis of the EGFL7 protein in WT, KO, and transfectants of EG7 (A), Colon-26 (B) by Anti -mouse VE-Statin Isoform 1 antibody.
